# Postal recruitment and consent obtainment from index cases of narcolepsy

**DOI:** 10.1186/s12910-016-0089-1

**Published:** 2016-01-16

**Authors:** Gambo Aliyu, Salah M. Mahmud

**Affiliations:** Vaccine and Drug Evaluation Centre, Community Health Sciences, University of Manitoba, Winnipeg, MB Canada

**Keywords:** Postal informed consent, Recruitment, Response rate, Cost

## Abstract

**Background:**

Access to research volunteers may be hampered by low numbers of cases and few eligible participants for rare diseases in clinical settings.

**Methods:**

We recruited volunteers and obtained informed consent by mail from narcolepsy cases in a case-control study, and here in we report feasibility, response rate, timeliness and cost. We invited index cases into the study by mail through their care-giving physicians then mailed study information and consent forms to cases that indicated interest in the study.

**Results:**

Of the 33 index cases invited, 15 (45.0 %) expressed interest in the study, and of those, 14 (93.3 %) returned their signed informed consents by mail. The median number of days from invitation to consent return was 39, interquartile range = 45, and the cost per consent obtained from the recruited subjects was $ 23.61.

**Conclusion:**

In this setting, postal recruitment for biomedical research on rare conditions is feasible and time and cost effective.

## Background

Research volunteers or authorized third parties acting on their behalves are required to provide informed consents with clear understanding of the research purpose, potential risks and benefits. Ensuring that decisions for participation in research are informed and voluntary, neither deceived nor coerced is mandatory [1–3]. Important elements of valid consent include information on the study purpose, procedures, potential risks and benefits of participation, confidentiality of health information, and the right to decline or reverse decision to participate at any time without any loss of benefit [4, 5]. This helps to guarantee autonomy of the research volunteer, a fundamental ethical requirement for using human subjects in research [6, 7].

Informed consents for research are usually obtained through a face-to-face discussion of the research with the investigator or study staff. Direct discussion with research volunteers is reported to improve understanding of the basic elements of research consent [8, 9]. In settings with low literacy levels, the use of multimedia, audio-visual illustrations in addition to the face-to-face conversation enhanced volunteers’ understanding of risks (adverse events) and voluntariness of the research [10]. However, in settings with high literacy levels research subjects with more formal education and literacy skills are more likely to understand the content of the consent including information on confidentiality and privacy protection [11].

Volunteers’ abilities to read and write were effectively utilized to obtain postal consents for access to their personal and confidential medical records [12, 13]. This study evaluates postal recruitment and informed consent obtainment for research involving a rare disease to assess feasibility, response rate, timeliness and cost in the context of the disease and setting where physical access to research volunteers for recruitment may be a challenge.

## Methods

Narcolepsy is a rare sleep disorder that can affect a person’s quality of life. The study objective was to evaluate factors associated with narcolepsy in the province of Manitoba, Canada as part of a wider search for evidence on the role of exposure to a pandemic influenza vaccine in the development of the disease. To achieve this, a retrospective case-control study was designed. Cases were defined on the basis of confirmation of clinical diagnosis of narcolepsy within the study period. A range of identified information including personal identifiers, symptoms of narcolepsy, diagnostic laboratory tests and their outcomes was needed. It was therefore necessary to first approach the narcolepsy index cases to determine if they were willing to participate, and provide consent to review their medical records for eligibility ascertainment followed by facility based one-to-one interviews of the eligible cases. The protocol for this study was reviewed and approved by the Research Ethics Board of the University of Manitoba.

### Postal enrolment

Routine clinic follow-up visits for narcolepsy patients could be several months or years apart particularly for cases with good clinical response to behavioral interventions. Targeting patients during routine clinic visits for the purpose of enrolment in research that does not involved prospective follow up of cases would be inefficient. We developed a list of narcolepsy cases from the two provincial sleep centers, one at the Psych and Sleep Disorder Center of the Health Sciences Center and the other at Misericordia Hospital, both located in Winnipeg, Manitoba. The cases were invited to the study through personalized mail correspondence from their care-giving physicians. The first invitation (letter 1) was written to a batch of 21 (Group A_1_) index cases. Interested persons were requested ***to call a phone number or send an email*** to express their interest in the study. After monitoring the response pattern, a second invitation (letter 2) which was a modified version of the first was mailed to another batch consisting of all non-responders to letter 1 (Group A_2_), and additional cases not included in the first invitation (Group B). This time all invited cases were requested to indicate their interest or lack of it by ***ticking one of the boxes in an expression slip*** and return it in the mail via a stamped envelope (included in the package). Non-responders to letter 2 in Group B were not sent a follow up invitation due to time limitations. The contents of the first and second invitation letters essentially differed in the final paragraph with the addition of the expression slip to letter 2 for the participant to pick an option and return in the mail. Letter 1 ended as follows:

“If you are interested in learning more about this study or have any questions you may contact SS, of the University of Manitoba research team at (204)272-xxxx or you can e-mail us at epimb@med.umanitoba.ca. Please note that you do not have to respond if you are not interested in participating in this study. Thank you for your time and consideration”

The concluding paragraph of Letter 2 reads:

“If you are interested in learning more about the Manitoba narcolepsy study, please contact SS of the research team at The University of Manitoba at (204) 272-xxxx. Even if you are not interested in learning more about the study, the research team will appreciate receiving a response from you indicating your decision by checking one of the boxes below and returning via the stamped envelope attached. Thank you for your time and consideration”.

The expression slip carried the participant’s assigned study number and boxes for the following options: interested, not interested and undecided.

### Consent obtainment

We mailed personalized packages to interested cases containing the study information, informed consent document and stamped return envelopes. The single page study information provided participants with a brief information on narcolepsy and why we wanted to know more about the disease, participant’s expected role in the study which include visit to the study center for direct interview and assurances of strict confidentiality of personal information in accordance with the law. The three page consent document provided details on the study purpose, procedures, potential risks and benefits of participation, and the right to decline participation. Confidentiality statements and contact information for questions or concerns about individual’s right as research participant were also included. Participants were requested to specifically indicate whether they agree or not to a range of confidential data to be extracted. Participants were requested to initial each page and append their name and signature on the last page. For children, an additional space was provided for their parent or legal guardian’s signature.

We measured the time taken for consent obtainment in days from the date we mailed the invitation to which the respondent indicated interest in the study to the date of return of signed consent. The cost of the consents obtained was estimated by tabulating the cost of the basic components: printed pages, postal stamps and staff work time in hours for packaging the invitation letters and consent documents, dropping and retrieval of responses from the office mail box. The in-kind contribution of the University’s official envelope, office space, desktop computer and other accessories were valued based on the applicable commercial prices or lease value, in the case of office space and included as a recurrent cost. Cost per consent obtained from recruited volunteer was calculated by dividing the cost of individual items with the number of consents obtained. These were then added to provide an estimate per consent obtained from a recruited volunteer.

## Results

**Fig. 1:**
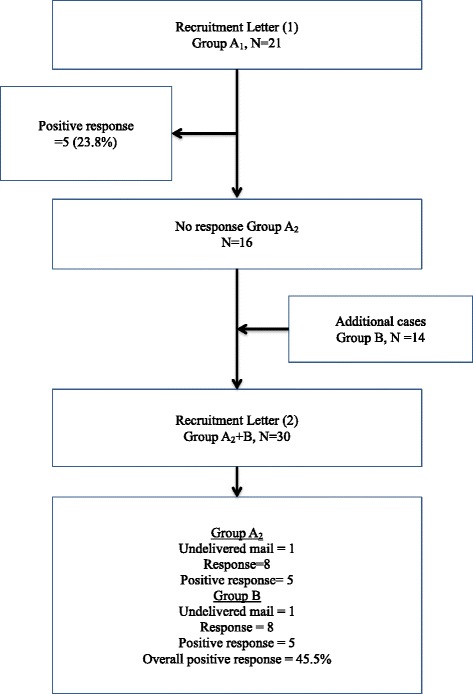
Postal Recruitment Flow Chart

A total of 33 index cases were invited in 51 personalized invitation letters, 15 (45.0 %) indicated interest in the study and 14 (42.4 %) provided written consent. Of the first 21 cases (Group A_1_) invited, 5(23.8 %) indicated interest via phone calls to the number provided in the invitation Letter 1. Among the 16 non-responders (Group A_2_) who were re-invited, a mailing was returned because the patient moved while 8 (50.0 %) returned their expression slips in the mail. Five (33.3 %) indicated interest in the study. Likewise, of the 14 freshly invited cases (Group B) through Letter 2, a mailing was returned as the person no longer resides in the address, 8 (61.5 %) returned their expression slips. Of the fresh invitees, 5 (38.5 %) indicated interest in the study. Figure 1 provides a flow chart for the postal recruitment and consent obtainment.

**Fig. 2:**
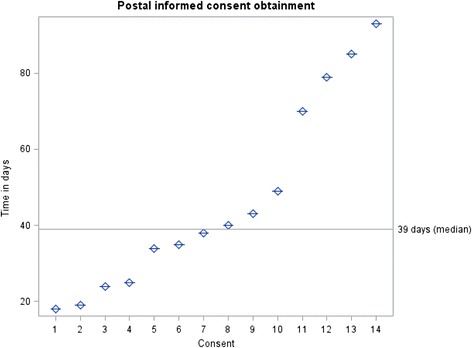
Time in days from initial invitation to obtainment of 14 informed consent documents

Altogether, of the 15 index cases that indicated interest 14 (93.3 %) returned their signed informed consents in the mail. All those who provided consent agreed to the range of confidential information to be extracted. The time in days from invitation to receiving the signed consents in the mail varies from a minimum of 18 days to a maximum of 93 days. The median time for consent obtainment was 39 days with an interquartile range (IQR) of 45 days (Fig. 2). In two cases, the consent forms were re-mailed because the initial documents they received were lost. These cases were identified because their signed consents were not returned and had to be contacted by phone to ascertain the status of their consent documents.

The estimated cost in Canadian Dollar was based on the components used from the initial invitation to consent obtainment which included: 31 postal stamps, 26 small size envelopes, 5 large size envelopes and 41 printed pages for the first invitation; 125 postal stamps, 45 small envelopes, 45 large envelopes and 90 printed pages used for the second invitation. It took on the average 5 to 7 min to print an invitation letter; seal, label, append stamp and drop in the office mail box. The estimated costs of the units of ingredients involved were provided along with corresponding costs per consent in Table 1. Most of the cost was accounted for by staff salary and to a lesser extent, the postal stamp.

**Table 1:** Components, cost and sources for the postal recruitment and consent obtainment

Components List	Unit	Quantity used	Unit cost (CAD$)	Cost per consent (CAD$)	Source
Paper page print	1	131	.10	0.94	Office manager
Envelope Size 5¾ × 9½ inches	6	71	.38	1.93	Canada post
Envelope Size 10 × 13 in.	6	50	.42	1.50	Canada post
Postal stamp	10	111	.85	6.74	Canada post
Staff salary	Per hour	6.45	26.04	11.99	Office manager
Office and accessories	Per hour	6.45	1.11	0.51	Office manager
Total				23.61	

## Discussion

Our study shows that postal recruitment of volunteers and consent obtainment for biomedical research is feasible and a good alternative in settings with efficient postal services, and in situations where face-to-face enrolment of research volunteers may be a challenge. Out of the 68 mail outs only two were undelivered and reasons where given for the delivery failures. The response pattern shows a relatively higher response when subjects were invited to respond through an expression slip and return in a stamped envelope than when phone calls or emails were used although we did not find this difference of statistical significance perhaps due to the small sample size. However, it shows the faith research volunteers have in the mail system in this setting. Investigators may wish to explore this volunteers’ readiness for postal enrolment for research in which the risks of harm or exploitation are minimal.

Observed response rate in this study may have been influenced by a number of factors including follow up invitation to the non-responders, use of personalized mails, stamped return envelopes and use of the University official envelope all of which were shown to increase response rate in postal surveys [14]. The response rate could have been higher if the five non-responders in group B were re-contacted. This shows that failure to provide initial response may not necessarily indicate a lack of interest as half of the re-invited subjects (Group A_2)_ provided responses. Some subjects may not find time to respond to the initial invitation. A reminder could make a big difference.

Reported studies differ on whether contacting respondents before sending surveys or consent documents increases response rate [14, 15]. Our findings suggest a strong correlation between acceptance of prior invitation to participate and subsequent return of signed consent in the mail. All except one of the participants who indicated interest in the study returned their signed consent. Willingness of the subjects to provide responses may be linked to the fact that narcolepsy could significantly affect a person’s quality of life and affected patients may be interested in studies that could aid understanding of the possible causes of the disease.

The median response time of 39 days is comparable to response time for mail surveys where respondents received postal questionnaires, and follow ups were sent to non-responders. Raziono and colleagues compared response times for postal and electronic surveys; the median response time for the postal survey was 33 days [16]. In this study, consent was obtained only from volunteers who indicated prior interest. The response time may have been less if the consent forms were sent together with the letters of invitation. However, the direction to which such approach would have influenced the response rate in this study is not known. Requesting the volunteers to respond within specific time frames of receiving the invitation letters could have also reduced wait time between invitation and subsequent consent mail out to improve response time.

For routine surveys, the electronic mail (e-mail) approach has shorter response times and costs less but the response rate may be low compared to the postal approach [16]. With increasing access, popularity and acceptability of internet based agreements and cyber security; debate on how best this platform can be used to obtain research informed consent should be encouraged. At the moment, the use of this platform is limited by several ethical challenges including privacy; confidentiality and risk of harm in addition to difficulties with regulatory oversights.

Assurance of privacy and confidentiality in online consent obtainment will be difficult since researchers do not have control over public cyber space and unlike the sealed mailing envelopes used to deliver postal consents, electronic mails are at risk of interception by a third party with no one to hold responsible even if such breach in confidentiality becomes apparent. The postal service helps to authenticate participant’s identity and physical presences as evidenced by the return of mails sent to previous residents. Similarly, people regularly change e-mail addresses and may not necessarily retain linkages to these addresses and document sent to them will automatically be lost or may be intercepted. The use of internet for consent obtainment will require respondents to have access to the internet, computers and possess computer literacy skills. The samples drawn are less likely to be representative and the findings may only be generalized to internet users.

The cost of consent obtainment in our study is higher compared to the 10.5 USD (13.9 CAD) reported by Raziono et al [16] and 8.7 to 13.2 EUR (13.0 to 19.72 CAD) from different approaches to postal consent obtainment evaluated by Christina Stenhammar and colleagues [17] . The cost per consent obtained is dependent on the response rate which was high (77 %) for the Raziono et al. and 30 % for that reported by Christina Stenhammar and colleagues. Increasing the number of consents obtained from 14 to 20 in this study will reduce the cost per consent to 18.33 CAD (22 % decrease) while lowering the response rate from 14 to 10 raises the cost per consent to 33.06 CAD (40 % increase).

## Conclusion

In conclusion, the acceptability of a postal approach to recruitment and consent obtainment demonstrates trust and confidence volunteers have in the system and its perfect alignment with the net users’ culture in this setting. It is also time and cost effective in situations where postal approach appears to be more effective in reaching out to a representative sample of volunteers within the study time frame. However, whether postal consent is ethically sufficient for research with greater than minimal risks of harm such as injuries, organ damage or even death is subject to debate.

## Abbreviations

CAD: Canadian Dollar
EUR: Euro
IQR: Interquartile range
SS: Study staff
USD: United States Dollar
